# Young medical doctors’ perspectives on professionalism: a qualitative study conducted in public hospitals in Pakistan

**DOI:** 10.1186/s12913-020-05681-w

**Published:** 2020-09-10

**Authors:** Aisha Jalil, Qaisar Khalid Mahmood, Florian Fischer

**Affiliations:** 1 School of Integrated Social Sciences, University of Lahore, Lahore, Pakistan; 2 Department of Sociology, International Islamic University, Islamabad, Pakistan; 3 Institute of Public Health, Charité – Universitätsmedizin Berlin, Berlin, Germany; 4 Institute of Gerontological Health Services and Nursing Research, Ravensburg-Weingarten University of Applied Sciences, Weingarten, Germany

**Keywords:** Medical curriculum, Physician, Doctor, Competence, Pakistan

## Abstract

**Background:**

Professionalism is amongst the major dimensions determining the competence of medical doctors. Poor professionalism affects the overall outcome of healthcare services. This study explores the perspectives of young medical doctors on professionalism in Pakistan.

**Methods:**

A qualitative study based on in-depth interviews was conducted with 60 young medical doctors, aged less than 40 years, who had studied medicine in Pakistani universities, were Pakistani nationals, and were employed at various hierarchical levels, from house officer to consultant specialist, in public tertiary hospitals in Pakistan. The respondents were identified through a multistage maximum heterogeneity sampling strategy. A semi-structured interview guide was developed based on a previous extensive literature review. Written consent was obtained from the hospitals and study participants. Qualitative thematic analysis was applied to analyse the data.

**Results:**

The data analysis revealed that rigidity of opinions, unacceptability of contrasting perspectives, false pride, and perceived superiority over other professions and patients were major components of poor medical professionalism. Most of the young doctors believed that there is no need to include professionalism and humanity course modules in the medical curriculum, because topics related to social sciences are deemed irrelevant to medicine and judged to be common sense. The doctors recognised good professionalism in themselves, while reporting unprofessional behaviour demonstrated by their colleagues and paramedics. Other factors contributing to poor medical professionalism included the use of social media applications during duty hours, ridiculing patients, substance use such as smoking cigarettes in the office, referrals of complicated cases to other hospitals, freeing up beds before holidays, lack of cooperation from paramedical staff, and inadequate role models.

**Conclusions:**

Poor medical professionalism among young doctors needs to be addressed by policymakers. There is a need to revisit the medical curriculum to strengthen professionalism. It is essential to develop the qualities of tolerance, teachability, and acceptance in doctors in order to facilitate interprofessional collaborations and avoid medical errors.

## Background

Medical professionalism is an essential component of a doctor’s toolkit, comprising technical expertise, interpersonal skills, time management, appropriate conduct, and medical competence. Clinical excellence cannot be achieved unless a medical doctor has impeccable personal values, such as altruism, empathy, respect for human life, and integrity [1]. Professionalism is fundamentally defined as a set of attitudes and behaviours that serve as the foundation for services rendered by an individual to society [2]. The construction of an inappropriate professional identity among doctors is a serious threat to ethical medical practice and the overall quality of healthcare. Every profession has a set of ethics grounded in the functions it performs. We define medical professionalism as a belief system in which doctors promise in their work to uphold an adherence to ethical principles and respect for human diversity [3, 4].

The American Board of Internal Medicine has identified seven universal components of medical professionalism: excellence, empathy, self-denial, accountability, duty, rectitude, and respect [5]. Empathy towards a patient is the most important after excellence and may be defined as the ability to understand the patient’s perspective [6]. Empirical evidence demonstrates that a lower sense of empathy leads to doctors’ malpractice, which is learned from the behaviour of seniors and training for impersonal associations with patient subjects during their time at medical school [7].

A study conducted on the poor management of human resources in healthcare demonstrated that the demotivation of healthcare providers was associated with poor management skills in the supervisor, conflicting relationships with colleagues, and lack of social support within the organisation [8].

International studies on medical professionalism have shown that professionalism is an important quality when identifying a ‘good doctor’, as well as their technical and communication skills [9, 10]. A study conducted in the USA assessed the perceptions of medical professionalism among medical students undergoing pre-clerkship, and highlighted attributes such as self-management, dependability, impartiality, responsibility, reputation, and ethics. In addition, it highlighted that the teaching in medical schools is an important determinant of medical professionalism [11]. Studies conducted in China have demonstrated that a lack of medical professionalism is connected to irresponsibility, procedural errors, conflict within medical teams, and financial concerns [12]. In Saudi Arabia, professionalism among the medical students and faculty of a medical university were compared. Within this study, the importance of academic integrity and of teaching and learning medical professionalism was stressed [13].

The fundamental spectrum of attributes of medical professionalism consists of three levels: individual, doctor–patient, and community [14]. Healthcare organisations strive to strengthen these essential attributes at all three levels. Professionalism is the built-in component of a doctor’s identity, which is integrated into the medical training phase. Scholars have been devising methods of improving the professionalism of doctors through teaching additional courses as part of the formal curriculum and introducing training sessions on humanism in medicine. Klemenc-Ketis and Kersnik [15] have experimented with the use of movies to increase the level of professionalism among medical students by depicting ideal situations involving positive behaviour and scenarios of rich resource settings. Other interventions used the role model and interactive method, in which the leaders of a healthcare organisation are aware of the cognitive importance of professionalism and demonstrate it in their practice [16]. Moreover, programmes for teaching the rich history and sophistication of clinical excellence were found to be effective in the teaching and learning of professionalism [17, 18].

### Pakistani context

Health services in high-income countries are continuously progressing away from a traditionally paternalistic approach towards a more patient-centred and egalitarian, collaborative approach to the doctor–patient relationship [19]. However, medical doctors in Pakistan have been resistant to accepting the validity of modern approaches to the doctor–patient relationship [20], which may be a factor in poor medical professionalism. There has been some debate about the quality of medical graduates over the last two decades and the medical professionalism of young doctors serving in both public and private-sector hospitals in Pakistan [9], but very few studies have focused on medical professionalism [21]. More attention has been paid to the qualities of a good doctor from a patient’s perspective in Pakistan, rather than malpractice [22]. According to a previous study, the medical community in Pakistan is attempting to address the deficiencies in professionalism by means of conservation, research, teaching, and evaluation [23].

Young medical graduates have socially constructed attitudes of superiority over other professions, perhaps because only the highest-performing students in secondary and higher secondary education become doctors in Pakistan. High-performing children generate a great deal of familial pride when they succeed in qualifying for medical school. Many financial assets, time, and energies are invested by their families, which puts pressure on the students competing to qualify for the limited places in medical colleges. These contextual factors engender a sense of superiority among the successful candidates.

The behavioural issues common among doctors are linked to the absence of a professional desire to serve humanity, a lack of contemplation about the subjectivity of human nature, and breaches of the Hippocratic Oath [24]. In addition, there are structural problems such as long working hours, low salaries, and unfavourable work environments that adversely affect medical professionalism. The previously high opinion of the medical profession among patients is perhaps being lost. Patients do not endow doctors with titles such as ‘life saver’, rather they call them ‘butchers’ who perform experiments on poor people [20]. In high-income countries, medical graduates are well trained in using human and professional skills to deal with their patients in an empathic manner [25]. However, there is a huge variation in the standards of professionalism between high-income and low-income countries [26, 27].

The struggling doctors in Pakistan are protesting peacefully about the causes of low salaries, hectic work schedules, and job insecurity. Abbasi [27] emphasised that the problems faced by doctors adversely affect the experiences of their patients and lead to the departure of highly capable medical doctors from Pakistan. The prolonged suffering, potential loss of health, lack of job security, unpaid service, and non-recognition of services rendered are the factors associated with strikes by young doctors [28, 29]. Any act of malpractice damages public trust in the medical profession and affects the quality of the physician–patient relationship. Patient-centred healthcare service delivery refers to an increasing concern for patient satisfaction and forms the foundation of medical professionalism in this era. The new values of medical professionalism and the worth of the patient as an equal need to be emphasised to a greater extent in the training of medical students.

This study provides a lens through which to examine medical professionalism from the perspective of young doctors themselves. Our work has international relevance because the contextual factors of unprofessional medical behaviour have not been openly discussed in previous research. Like other areas of public health, professionalism has also not been extensively explored in Pakistan. Only a few studies have examined professionalism among medical students in Pakistan [17], but no previous work is available that examines professional behaviour among young doctors of purely Pakistani heritage. In order to be able to devise a solution to impaired professionalism, it is important to understand what constitutes medical professionalism in a given context. Thus, this study aims to explore the perceptions of professionalism among young medical doctors, and the facilitators and barriers to professional behaviour. Thus, we focused on the following research questions: How professional do the doctors think they are? How do they perceive that the structures, processes, and outcomes of medical care affect their medical professionalism? What is the nature of the problems facing doctors that hinder their ethical medical practice?

## Methods

### Study design

Since the purpose of this study was to understand medical professionalism among young medical doctors of Pakistani heritage, an interpretive ontological and epistemological stance was adopted [30]. An exploratory qualitative study was conducted, because an understanding of contextual and normative factors is best attained through in-depth interviews.

### Participants

Participants were identified using a multistage maximum variation sampling method [31, 32]. We had to contact 64 medical doctors to reach the a priori defined sample size of 60 participants, illustrating a high response rate of 93.8%. In the first stage, the hospitals were selected, based on the criterion that the largest public-sector teaching hospitals in three regions within Pakistan (Sindh, Punjab, and the State of Azad Jammu & Kashmir) be included. Thus, all the major public-sector, tertiary-care hospitals were sent written requests explaining the purpose of the present research. The study was carried out in the consenting hospitals. In the second stage, criteria sampling was conducted to recruit participants who were young doctors (less than 40 years of age), in the early years of their professional careers, who were working at various hierarchical levels as house officers, medical officers, trainees, senior registrars, and specialists in various specialities and departments (Table 1). Doctors of physiotherapy, dentistry, or nutrition were not recruited for this study. We focused on medical doctors only, who had obtained an undergraduate, first professional degree in medicine (MBBS) from Pakistani universities. Maximum variation sampling was used to recruit doctors of all possible ranks deputed in various departments of the selected hospitals.

**Table 1:** Multistage maximum heterogeneity sample calculation

Sampling stage 1	Selection of hospitals	Criteria: Largest public-sector teaching hospital in provinces of Sindh, Punjab and the State of Azad Jammu & Kashmir (AJ&K)	1 hospital in Punjab 1 hospital in Sindh 1 hospital in AJ&K
Sampling stage 2	Recruitment of doctors	Criteria: (i) Age: 24 to 32 years / 33 to 40 years (ii) Gender: male / female (iii) Service level: house officer, medical officer, PGT trainee, senior registrar, specialist	Calculating factor: 2 × 2 × 5 = 20 doctors selected in each hospital

### Interview guide

A semi-structured interview guide was developed based on an extensive literature review. The respondents’ profile attributes included: gender, age, nationality, qualifications, designation, area of specialisation, and length of service. The interview guide included open-ended questions concerning: reason for becoming a doctor; hardships and gratification; understanding of the meaning of professionalism in medicine and the qualities of ideal medical doctors; young doctors’ perceptions of patients (ridiculing the patient, treating them as subjects, justified misbehaviour); perceptions of flawed technical expertise (procedural errors, absence of physical examination, and inability to diagnose without pathology tests); dealing strategically with security issues and safety at work; personality traits (patience, tolerance, flexibility, altruism, service to humanity, high moral standards, integrity, honesty, and duty); process and structural factors hindering professionalism; self-pride and shaming of other professions; and what needs to be done to improve the situation (Additional file 1).

### Ethical approval

Participants signed a written consent form before the interviews were conducted. This consent form was developed using the World Health Organisation’s checklist for English-language consent forms for health services. The methodology of this study was approved by the Institutional Review Board of the International Islamic University, Islamabad. Written permission was obtained from the hospitals where the study was conducted. Since the phenomenon under consideration is sensitive and could have serious implications for the respondents, neither the names of hospitals nor any information that could identify the doctors are disclosed in this paper.

### Data collection

The interview guide and respondents’ answers were in the English language. The interviews were conducted by the first author and a research assistant. The research assistant had prior experience of qualitative data collection and surveys in health settings and undertook pre-interview training. The doctors were interviewed in their offices during breaks and at the end of duty shifts, at their convenience. We ensured privacy during the interviews and data security after audio recording. Any information that might identify the doctor was not transcribed. Initially, the doctors gave socially desirable answers and were not willing to spend much time, perhaps due to busy work schedules or lack of interest in the subject matter of the research. Off-the-record statements and comments by doctors and researcher observations were noted for further usage in data analysis [33]. For each interview, we kept notes of field experience, observations, and important statements made by respondents after the recorder had been turned off. We sought verbal permission from the doctors before jotting down their statements. The data collection was completed during April and May 2019. The interview time ranged from 30 to 75 min. The verbatim transcription of the audio recordings was undertaken by the principal investigator and a research assistant.

### Data analysis

Data analysis took place after the completion of data collection. The data were analysed using qualitative thematic analysis. We followed the 32-item checklist *Consolidated Criteria for Reporting Qualitative Research* to report the findings of this research [34]. To ensure inter-rater reliability, the first author and research assistant each coded the data. Both researchers mutually discussed this coded data to compare notes and arrive at common and variant codes. The codes were then grouped into common themes. Finally, the themes were grouped into categories and relationship identification was performed. Additional file 2 exemplifies the codebook. Upon completion of the data analysis, the findings were taken back to three of the study participants, who had expressed their interest during the interviews, to ensure credibility.

## Results

Throughout the data, we found that the perceptions of young doctors demonstrated poor medical professionalism. Occasional self-comparison with doctors in previous generations implied a decline in the spirit of serving humanity. The findings are based on an analysis of the statements of participants and observations made by researchers during data collection. Overall, sixty participants were interviewed, with equal representation from each of the three selected regional hospitals. The social demographic characteristics of participants are presented in Table 2. Our data analysis indicates the following themes and categories relating to the perspectives of young doctors on medical professionalism, how their attitudes diverge from standards of professionalism, and the contextual factors affecting their medical professionalism in the public hospitals of Pakistan.

**Table 2:** Characteristics of participants

Characteristics	Punjab	Sindh	Azad Jammu & Kashmir
Age			
24 to 32 years	10	10	10
33 to 40 years	10	10	10
Gender			
Male	10	10	10
Female	10	10	10
Highest qualification^a^			
MBBS	4	4	4
FCPS1	4	4	4
FCPS2	4	4	4
Diploma	4	4	4
Others	4	4	4
Service level			
House officer	4	4	4
Medical officer	4	4	4
PG trainee	4	4	4
Senior registrar	4	4	4
Specialist	4	4	4
Service length^b^			
Less than 7 years	12	9	13
7 to 12 years	8	11	7
Department			
Outpatient	7	8	7
In-admission	6	6	7
Emergency and accidents	7	6	6

^a^ Qualified in Pakistan are selected. Doctor of Physiotherapy (DPT) and Bachelor of Dental Surgery (BDS) are not recruited

^b^ Average age of completing MBBS (MD) is 23 to 25. This experience includes one year of house job

### Flexibility, tolerance, and poor medical professionalism

The study participants demonstrated characteristics such as rigidity of opinion, inflexibility, and non-acceptance of contrasting perspectives. Some of the doctors became angry during the interview about the subject matter of the research, regarding it as a strategy to make doctors furious. The statement of one respondent may be useful at this point to further clarify the scenario: *“I don’t understand why you’re carrying out such research. This is not your field – you’re not a medical doctor. A doctor of medicine can ask us such questions.”* Another respondent said: *“Because of this type of research, doctors are unable to perform well on duty.”*

One study participant said about the need for tolerance:

I think to some extent tolerance is good, but it becomes negative if we use it everywhere, like we will forget to even react in situations where a reaction is actually needed. So, it has little to do with our field*.*

The young doctors believed that they are superior and that there should be no collaboration with experts in other fields. Only a medical doctor has the capacity to work with public-health-related matters. There is no need for the inclusion of a professionalism and humanity course in the medical school curriculum. The majority of respondents thought that social-science topics are irrelevant to medicine and are merely common-sense things that they already know. For example, a male medical officer said:

Everybody knows that medicine is difficult to study and only exceptional students are selected for medical colleges and universities. Other subjects are also useful for weak students, but medical science is at the top of the hierarchy of various study disciplines. Social sciences and arts are something we already know, and we do not need to study them as additional courses and I do not think ethics has much to do with our field. Healing or the patient is the most important*.*

Similarly, a male house officer said:

It is of no use to teach humanities. Then there is no point in teaching humanities to medical students. This skill will not be used anywhere. It is useless for us to waste time studying irrelevant subjects. Already medical students have more useful books to study extensively. Why waste time on a skill which is never going to be used in practice?

Individually, doctors reported that they themselves are highly professional, but that some other doctors demonstrate unprofessional behaviour. These reported characteristics include using social media applications during duty hours, ridiculing patients, and substance use such as smoking cigarettes.

### Perceived superiority and a paternalistic approach

We found that the perceived inferiority of other professions and patients were widely prevalent among the participants and led to the demonstration of false pride. Most participants reported that tolerance and acceptability have little to do with medical professionalism. One doctor said: *“A doctor is a ‘doctor’.”* All the participants believed that their knowledge of medicine has equipped doctors with a superior position over their patients and other professions. The belief that patients know nothing was common among all the participants. One female doctor said: *“A down-to-earth approach wouldn’t work for patients in Pakistan. Patients don’t mind the harsh conversation with the doctors, rather they think that a doctor who snubs is a competent doctor.”* Another respondent said: *“Patients know nothing, irrespective of the fact that he is educated or not – obviously, one would not become a doctor by googling diseases and symptoms.”* This statement reveals a sense of superiority over patients and other human beings. The doctors demonstrated a lack of any perceived need to establish congruence with patients.

### Training under positivism

Almost all the participants claimed medical professionalism in themselves while reporting other doctors as having distorted professional ethics. Their training under the positivist school of thought does not leave any room for subjective human nature. This is depicted in the words of our participants, who believe that humanities, ethics, and professionalism are common-sense phenomena and since medical doctors pass through the toughest educational screening and training, they do not need to learn these skills. There was reporting about clashes of opinion and petty issues among the doctors, which reveals rigidity and a lack of acceptance of other viewpoints, perhaps linked to their medical school training under scientific knowledge trends.

### Taking their duty seriously

As one example, we were told that medical doctors use their mobile phones while seeing patients. This is a common practice among doctors in Pakistan, which affects the quality of attention they give to patients. Furthermore, smoking and substance use by male doctors was also reported by both female and male doctors.

### Ridiculing the patient

There was reporting of sub-standard incidents of laughing at patients in which senior doctors discuss critical patients in debriefings with house officers. Making fun of people who are in pain and dependent for help on the doctors on duty does not imply medical professionalism. The words of one house officer clarify this finding:

I became a doctor to serve mankind and I have tried my best to do my duty honestly. I feel depressed when I see my colleague house officers ridiculing old patients admitted in critical condition. None of the house officers want any patient to expire in their bed so they refer patients to other public hospitals without treating them. Even it was depressing for me when I heard MO making fun of old patients in pain. (Probe: What did MO say while making fun? If you can recall?) Yes, he came in and asked his house officers: “How many elderly patients did you kill today?” And it was happening every day as a fun routine in meetings of house officers with MO*.*

### Inadequate role models

During the first few sessions, the doctors gave socially desirable answers, such as: “*We’re very professional and none of the doctors show aggressive behaviour on duty.”* However, other respondents emphasised that aggression is a basic part of human nature. In conflict situations, both parties are justified in demonstrating aggressive behaviour. Participants reported that their superiors and colleagues who are rude and critical and create conflict are just following human nature. None of the participants mentioned the need for anger or conflict management. Mistreatment by superiors and a high workload were reported by a few respondents. The senior staff should set good examples for young doctors to follow, said one respondent:

When they are not coming on time and insult patients on a single question, how can young doctors follow their trends? They have clashes with others. They criticise each other on disease management strategies – they couldn’t even agree on the dose of anaesthesia*.*

### Intermediary non-medical staff

Several practical issues were reported in relation to working in healthcare teams, and non-cooperation by paramedical and nursing staff. Treatment errors are linked with the non-cooperation of non-medical staff, especially nursing assistants, during the execution of medical procedures:

The government initiatives have made nurses superior to doctors by giving them job security and reasonable salaries that exceed what is paid to doctors. So now the nurses clash with doctors – even during operations they do not cooperate with us … Like during surgery when I asked for a retractor from nursing staff, she didn’t respond so I asked again. She kept standing silently and then I had to go further away and get it myself*.*

Due to the status of their job and the facilities in the public sector, nursing staff often clash with doctors. Technically, the doctor is head of a healthcare team, but the situation is different in public hospitals in Pakistan. Female doctors expressed their fear of abuse from nurses if they fail to make them happy and concern about provoking anger on petty issues:

*In gynae wards, it is very important for all house officers to have a good “Hello” or “Hi” with nurses, because if you annoy them and cross them, they will not do anything for you so we address them nicely as* baaji jee *(in Urdu), meaning elder respectable sister.*

Another female trainee doctor said: *“We have to call the nurse in a low and sweet tone so she may give us the thing that we need. All of the senior doctors even address the nurse as nurse* jee*”* (in Urdu, *jee* is used with names to show extreme respect). Participants reported that patients also complain about the non-cooperation of nurses. One of the doctors reported that she has heard a nurse putting off a patient by saying: *“I’m busy now – come tomorrow. I will tell you how to use insulin, diet chart and check blood sugar …*”.

### Ethical medical practice

There was inconsistency in the perceptions of doctors about what describes ethical medical practice. The majority asserted the importance of the technical aspects of medical care. We found few responses in favour of service to humanity, sacrifice, or honesty. Only one male trainee doctor spoke about what professionalism is: *“A good doctor sacrifices his sleep and appetite so that his patient can sleep well.”* Overall, only two doctors said that they are serving humanity and emphasised that the purpose of joining this profession was not the financial benefits:

How can people expect doctors to think of serving humanity when they are having financial problems at home? Their families also need money for survival and a good quality of life. Doctors should be highly paid, because they have worked harder than people in any other profession. For following standards of what is right and wrong, there has to be an overall workplace environment that will allow us do that*.*

### Need for accountability and training

The respondents agreed that a mechanism for accountability is required in hospitals, but emphasised the need to appoint a medical doctor with expertise in evaluation. This suggested that medical doctors would not like to be judged by a person with a non-medical background. Few mentioned issues of poor professionalism in general, but at the same time reported that training in the non-technical aspects of care (i.e. interpersonal communication and professionalism) in their degree programmes is not needed. They still viewed it as irrelevant content.

### ‘Teachable-ness’ of doctors

When disagreement with experts in other fields occurs, it is observed that doctors are not teachable and lack the ability to accept other viewpoints. Something is seriously lacking in their training at medical school. This can be understood clearly in the words of one respondent in this study, who said:

How can a social worker or a lawyer tell a doctor what to do in healthcare practice? The other professions are adopted by people of average intellectual and mental abilities. A doctor is a “doctor”. If something must be done about society or there is any kind of programme evaluation, healthcare should be assessed by a medical doctor. There is no need for social scientists and humanities here*.*

These statements demonstrate the irrational sense of personal value and false pride that doctors have in the medical profession. However, it can be understood only when contextualised within South Asian society. This attitude implies that doctors are not teachable and have no skills relating to tolerance or acceptance.

### Work in interprofessional collaborations and conflict management

There was no tendency to work in interprofessional collaborations because doctors felt that they did not need or trust the services of experts in other fields because such fields are chosen by low-performing students. This attitude is not professional in nature. The findings also implied that there are clashes of opinion within teams of doctors over prescribed treatments and procedures. The effectiveness of teamwork in healthcare is associated with patient outcomes. Respondents justified frequently being in conflict situations with other doctors and healthcare providers. One female doctor said: *“It is normal and healthy to get into arguments and conflict.”* Another said: *“We cannot avoid getting into conflict because it is natural and does no harm.”* Little concern about learning conflict management skills was found.

## Discussion

This study explores the perceptions of medical professionalism among 60 young doctors and provides a qualitative account of poor professionalism. Our results indicate that young doctors are low in professional ethics due to personal, procedural, and structural factors. We found that rigidity of opinions, lack of tolerance, inability to accept contrasting perspectives, false pride, and perceived superiority over other professions and patients were the major personality traits contributing to poor medical professionalism. Most of the young doctors believed that there is no need to include professionalism or humanities course modules in the medical curriculum, because topics related to social sciences were considered irrelevant to medicine and judged to be common sense. Doctors reported malpractice among others but reserved professionalism in themselves. Other factors contributing to poor medical professionalism included the use of social media applications during duty hours, ridiculing patients, substance use such as smoking cigarettes in the office, referrals of complicated cases to other hospitals, freeing up beds before holidays, lack of cooperation from the paramedical staff, and inadequate role models [35].

Although the profession cultivates humanism, the findings are quite contradictory and bring the quality of medical education in Pakistan into question. Medical professionalism is not taught explicitly at medical colleges or medical universities in Pakistan [36]. Medical students only learn professional values informally through the role-model method because the national curriculum for medical universities does not include courses on ethics or professionalism.

Like our findings, empirical studies in rich healthcare settings have also demonstrated the incidence of a significant degree of arrogance in medical doctors during their pre-clerkship [23, 37, 38]. In countries like Pakistan, parents always encourage their children to opt for medical science as a profession. This is due to misleading expectations about the opportunities of getting highly paid jobs and the social prestige attached to this profession. This social prestige also contributes to the false pride among young doctors. Parents invest heavily in their children’s education to make it possible for them to gain admission to medical science courses. Consequently, once a student secures admission to medical college, he/she and his/her family take pride in this achievement. In addition, medical colleges train their students to take pride in being more successful than students in other disciplines. However, after working hard for many years, they remain unpaid or underpaid [27].

In such a scenario, medical students feel themselves superior to others, although a low amount of money is spent on the healthcare sector, particularly for medicines. There is no previous empirical study that validates these contextual realities. Our respondents highlighted their struggles and the pressure from their families when they were gaining admission to medical universities. Addressing these attitude problems is essential because it manifests as malpractice.

Listening to the viewpoints of patients about their health helps doctors to uncover possible reasons for ailments, but doctors’ sense of superiority does not allow them to fully understand their patients’ condition [11, 12]. Moreover, high scorers can opt for medical schools, but the ability to diagnose with accuracy is a skill that is not possessed by all the medical doctors who graduate. Therefore, most young doctors do not achieve the standard of diagnosis that a successful doctor should have. Besides diagnostic skills, the success of healthcare procedures depends on cooperation between the entire healthcare team: doctors, nurses, and other non-medical staff [24]. Our respondents reported non-cooperative behaviour by the nursing and other non-medical staff [39, 40]. One possible reason for this is an inability to express concerns based on equality. However, contradictory evidence was also found about most of the doctors, who do not treat their subordinates appropriately [16, 25].

Doctors believe that good medical practice is hampered by external factors. Occasionally, this resulted from a comparison with medical doctors who had started practice three decades before and were provided with better opportunities and facilities. They also pointed out that senior doctors are deficient as role models because they criticise their juniors and disrespect them. There was a demonstration of dissatisfaction with both their role models and supporting paramedical staff. This supports previous studies, which have asserted the importance of learning from role models [8]. However, it is also important to be able to work in interprofessional and healthcare teams [31].

The lack of seriousness in doctors’ attitudes towards duty was linked with excessive mobile phone and internet use during duty hours, which is in line with previous research [41]. Furthermore, it is alarming that young doctors did not judge the use of mobile phones and the internet during duty hours as being detrimental to medical professionalism [42]. Other studies have also demonstrated that the use of Facebook and social networking blurs the professional boundary between doctor and patient [43, 44].

To provide better healthcare, it is necessary to train doctors in the skills that will make them successful professionals. We recommend the integration of some ethics, humanities, and social science courses into the national curriculum for medical students. Human identity formation is equally as important as the development of technical expertise. This is the responsibility of medical colleges [8]. One needs to consider that professionalism must be investigated as a culturally delicate phenomenon. Therefore, context-specific, competence-based training for medical students should also be designed and implemented [45]. Both practice and the medical education system should be restructured to adopt international standards of medical professionalism [46]. Thus, learning about patient-centred medical practice is a prerequisite.

### Limitations and strengths

There were certain limitations to this study. Previous research conducted in Pakistan has applied quantitative methods to suggest that medical professionalism is high among medical students [21, 22]. In contrast, the present study did not measure professionalism quantitatively. Our findings may not be representative for Pakistan, because the study was conducted in only one province. The effects of sample selection and response biases may have influenced our findings.

However, we attempted to avoid biases in designing the interview guide, data collection, analysis, and reporting. The interview guide was developed based on concepts that emerged from the literature review. The context-based questions were phrased formally and specifically. The interview guide did not include any suggestive or pushy questions. The data collection, transcription, and analysis were performed by two researchers. In reporting the findings, direct quotes from participants were cited. The interviewers did not react to critical or aggressive responses in order to avoid interviewer bias.

Future studies could develop a comprehensive scale to measure medical professionalism. Prospective research should focus on developing strategies and methods for improving professionalism. How medical professionalism increases when young Pakistani doctors seek jobs in high-income countries should also be analysed.

## Conclusion

Our findings demonstrate poor medical professionalism among young medical doctors in public hospitals in Pakistan. This was indicated by false pride, lack of willingness to work in interprofessional collaborations, shaming other professions, a perceived sense of superiority over patients, ridiculing patients, the use of mobile phones and social media at work, and medical errors. These issues need to be addressed through systemic changes, particularly by revisiting the medical curriculum and recasting it in a more humanistic way. Better role models highlighting the qualities of tolerance, teachability, and acceptance may raise the standards of practice.

## Supplementary information

**Additional file 1.** Questionnaire.

**Additional file 2.** Exemplar codebook.

## Data Availability

Pseudonymized transcripts are available from the corresponding author upon reasonable request.
