# Supplementary material for: Young medical doctors’ perspectives on professionalism: a qualitative study conducted in public hospitals in Pakistan
Source: BMC Health Serv Res. 2020 Sep 10;20:847. doi: 10.1186/s12913-020-05681-w (PMC7488058; doi:10.1186/s12913-020-05681-w)
Supplement: Supplementary file 1 — Additional file 1. Questionnaire. [file 12913_2020_5681_MOESM1_ESM.pdf]

Interview Guide

Gender: F/ M Age: ________ Nationality: ______________
Designation: _________________ Qualification: _________________
Specialization: ____
Number of years of professional experience: ______________________________

Q1. Since how long you have been serving your profession?
Q2. Tell something about your journey of becoming a doctor? About the struggles and hardships.
Q3. When and how did you decide to become a medical doctor?
Q4. In your opinion, why people prefer becoming a medical doctor over other professions in Pakistani society?
Q5. Do you think that the parents force their children to go for this profession?
Q6. What was your purpose for becoming a doctor? To make money or to serve the poor patients?

I want to ask you some questions about what generally patients think about young doctors? Please feel free to share anything that comes to your mind in this regard.

Q7. Have you ever noticed your colleagues making fun of patients?
Q8. Have you ever seen other doctors discussing that the critical patients cannot be admitted for the fear of death on a duty doctor's bed? It has been happening in major tertiary care hospitals of Lahore and is the major reason behind referrals as per patient's opinion.
Q9. When is the rude behavior of doctors justified towards the patient and attendants?
Q10. Patients think that the doctors in training and house officers do experimentation on poor patients in public hospitals for their learning. What is your opinion in this regard?
Q11. The technical errors, mistreatment and inability to diagnose are the frequently reported factors of patient dissatisfaction? How do you perceive the situation?
Q12. Are there situations in which you encountered patients and attendants threatening you for things that were not in your control?
Q13. Why is the over-reliance on tests when the doctors who lived back in the three decades could diagnose without any pathology test or scanning aid?

Professionalism in medical doctor

Q14. What are the most important things that define professionalism of a doctor?
Q15. Why is it important for a patient to tell the doctor about who he is, to be treated nicely by the doctor?
Q16. What factors are hindering the doctors from professional medical practice?

These are the personality traits on which professionalism of a person is assessed:

Q17. Becoming a doctor was linked with the spirit of serving humanity and selflessness, what is your take on this.
Q18. How important is patience for a medical doctor?
Q19. In your opinion, do the doctors show tolerance for each other and experts of other professions while working in team?
Q20. To what extent flexibility is required in healthcare profession?
Q21. Does anything come to your mind when you think about high moral standards of a doctor?
Q22. How do the young doctors learn and use the sense integrity and honesty on their duty?
Q23. Please discuss those procedural and structural factors that hinder medical professionalism.
Q24. People say that doctors have self-pride and there is a common practice of shaming other professions. How true is this notion?
Q25. Please comment on the use of mobile phones in the hospitals.
Q26. Do you think that there should be a mechanism for monitoring and evaluating medical doctors conduct and competence in the hospitals?

Researcher observations: ____
