# Supplementary material for: Young medical doctors’ perspectives on professionalism: a qualitative study conducted in public hospitals in Pakistan
Source: BMC Health Serv Res. 2020 Sep 10;20:847. doi: 10.1186/s12913-020-05681-w (PMC7488058; doi:10.1186/s12913-020-05681-w)
Supplement: Supplementary file 2 — Additional file 2. Exemplar codebook. [file 12913_2020_5681_MOESM2_ESM.pdf]

Additional file 2: Exemplar codebook

Participant responses	Code	Code frequency	Themes	Categories
'See these rules like tolerance do not work in our society… if we are going to be polite… patients and attendants take it as our weakness and start repressing us.'<br>'I think to some extent tolerance is good, but it becomes negative if we use it everywhere like we will forget to even react on situations where reaction is actually needed… so it has less to do with our field…'	Extent of tolerance.<br>Felt that there is no need for acceptance and tolerance skills.<br>Patient abuse.<br>Friendly behaviour is a personality weakness.	13<br>39<br><br><br>45<br>28	Acceptability<br>Tolerance	Doctors on components of professionalism.<br>Young medical doctors described.<br>Absence of training on medical professionalism
'because of this kind of researches we cannot focus on our work… we do not need certificate of excellence from anyone… we are doing the most difficult job and this is a thankless nation… but your research has no benefit for doctors as well as patients…'<br>'A doctor is a doctor'<br>'how a person who is not a medical doctor can understand and study problems of doctors… it should be allowed to experts of equivalent qualification.'<br>'How can people expect from doctors to think of serving humanity when they are having financial problems at home… their families also need money for survival and a good quality of life… doctors should be highly paid because they have worked harder than people in any other profession…'<br>'Subjectivity in human nature is not something one needs to learn... It would be waste of time…'<br>'how will you know what doctors do when you are not a medical doctor!'	Expression of:<br>Anger<br>Disgust<br>False pride<br>Extracted Personality Traits:<br>Egotistic<br>Self-assertive<br>Inflexible<br>Judgemental<br>Lack of capability of being taught by experts other than medicine.	 6<br>51<br>55<br><br><br>57<br>48<br>56<br>38<br>41	Non acceptance of other viewpoints.<br>Working in healthcare teams.<br>Shaming other professions.<br>Non-willingness to learn.<br>Absence of expression management training.	Depreciated value of other professions.<br>Perceived superiority of medical profession.<br>Perceived inferiority of patients.<br>*Teach-ableness*
'Senior doctors have better facilities then us… they are working on more than one position… like teaching and consultation at one and the same time with administrative responsibilities…'	Bad examples set by Seniors on behaviour with patient, time management,	34	Gap<br>Role model method<br>Facilities for specialists and senior doctors	low professionalism<br>Time-based comparison

'There situation is different from us…'<br>'When they are not coming on time and insult patient on single question, how can young doctors follow their trends! They have clashes with others… they criticize each other on disease management strategies… even wouldn't agree on the doze of anaesthesia'<br>If you are saying that the doctors three decades ago could diagnose better without relying on radiology and pathology tests. then your hypothesis is flawed because at that time there were more deaths resulting inaccurate diagnosis… mere estimates about an illness possess chances of error so the tests are for benefit of patient.'	teamwork,<br>procedural errors			
'I don't think medical doctors need additional courses to take on ethics and professionalism because these are common sense things that everyone already knows…'<br>'humanities are subjects for low achieving students…'<br>'a person of medicine can monitor and evaluate performance of doctors... not the ordinary social worker or something…'	Belief that professionalism and humanism are common sense phenomenon.	49	Actual need for teaching ethics, humanities and medical professionalism<br>Need for monitoring & evaluation	Loopholes in formal curriculum<br>Teaching and learning of medical professionalism<br>Train doctors under tradition of positivism
'we are multitasking people...'<br>'Others might be using cell phones, but I don't do that…'<br>'This is inevitable… you know there is not a single doctor who is not using phone at work… our seniors do that so this cannot be expected from juniors…'<br>'This is happening even in private clinics also…patients do not mind…'	Use of mobile phone during interview and while researcher was talking to them.<br>Use of social media apps during work hours.<br>Ineffective and unrelatable role models.	17<br><br><br><br>12<br><br><br>31	Mobile phone and social media usage on duty.<br>Ineffective role models.	Seriousness towards duty
'Male doctors smoke in their collective office and administration does nothing about this…'<br>'They say that there smoking is not affecting their performance at work…'<br>'It is a matter of personal choice…'	Smoking<br>Substance use<br>Ineffective and unrelatable role models.	9<br>1<br>6	Lack of control on unhealthy practices	Component of Medical Professionalism<br>Social responsibility

I became a doctor to serve mankind and I have tried my best to do my duty honestly… I feel depressed when I see my colleague house officers ridiculing old patients admitted in critical condition… none of the house officers want any patient to expire on their bed so they refer patients to other public hospitals without treating… even it was depressing for me when I heard MO making fun of old patients in pain.. (Probe: what did MO say while making fun? If you can recall) … Yes he came in and asked his HOs: [in Urdu: '*haan bai aj kitne babay ludhakaay tum logun ne'*] meaning: how many elderly patients do you kill today? And it was happening everyday as fun routine in meeting of HOs with MO…'	Make fun of the patients.<br>Not taking death as a serious matter.<br>Fearing death on patient on bed assigned.<br>Referrals of complicated cases.<br>Conflicting healthcare teams.	2<br>5<br>23<br>4<br>9	Ridiculing patients.<br>Working in healthcare teams.<br>Referrals.	Effects on Patients
'I don't understand that how a poor and illiterate patient can tell about the competence of a doctor…'<br>'Professional doctor is the one who listens to his patient's complain, behaves well and examine nicely… I think all of the doctors work professionally...'<br>'we do symptomatic treatment and healing is not in our control…'<br>Medical professionalism is only something related to services provided by us and the professionalism standards for other professions would not be applied on this field…<br>'…Even our MOs do not know many of the things and make incorrect diagnosis…'<br>'diagnostic tests are part of diagnostic process and for history of patients health… we cannot get to know the problem unless…'<br>'Educated people do texts every 6 months for their personal satisfaction but in this hospital, patient's don't appreciate this…'<br>'Yes, it is happening (over reliance on tests), but unnecessary tests are not good it creates more depression.'	Ability to diagnose.<br>Treatment plan effectiveness.<br>Listening to patient.<br>Ethical behaviour.<br>Patients do not understand technicalities and blame doctors for what is not in their control.<br>Doctors do not have command on diagnosis.<br>Test reliance important	31<br>28<br>34<br>39<br>54<br>10<br>54	Technical expertise<br>Patient comprehension<br>Competence<br>Conduct	Procedural factors
'we cannot smile and be frank with patients who are unknown to us and generally it is not considered	Friendliness and being nice.	35	Communicational behaviours	Interpersonal factors

respectful to smile at and be frank with people in our society…'<br>'Being polite with patients depends on the circumstances... sometimes we must be aggressive to make them understand… we have short time and it is not possible to repeat the same thing again and again'<br>'It depends person to person, ideally there should no discrimination of patients based on age gender race and religion. This is prohibited in medical ethics…'<br>'I do agree with this point, doctors mostly judge people with appearance and behave friendly with patients on personal liking...'	Social meaning of smile.<br>Qualities of good behaviour towards patient.<br>Functions of good doctor.<br>Shortness of time available.<br>Equality based treatment of patients of all social classes.<br>Patient physical appearance.	11<br>52<br>47<br>53<br>31<br>19		
'…The government initiatives have made the nurses superior to the doctors by giving them job security and reasonable salaries that exceed what is paid to the doctors… so now the nurses clash with doctors... even during operations they do not cooperate with us… like during surgery that I asked for retractor from nursing staff, she didn't respond so I asked again she kept standing silently and then I had to go further away and get it myself…'<br>'In gynae wards, it is very important for all HOs to have good hello hi with nurses because if you annoy them and cross them… they will not do anything for you so we call them nicely as *baaji jee (*in Urdu*)* meaning elder respectable sister'<br>'patients will suffer less, if doctors are not facilitated.'<br>'once spot of respect for everyone…'	Technical errors<br>Nursing staff<br>Team clashes<br>Doctors performance<br>Subordination to nurses when the head of medical team is a specialist doctor.<br>Work nature in Pakistani healthcare structure.	21<br>37<br>24<br>59<br>42<br>60	Nursing staff non-cooperation.	Structural hurdles.<br>Importance of working in teams.
'I want to guide them but every time I fail because of limited time, but I don't show disgust, as a doctor it's my duty to guide them.'<br>'We treat people 90% of poor, they have hygiene issues we try to sit little far from them so we can protect ourselves from viruses.'	Patient preference for mobile credit over buying soap.<br>Show disgust	16<br>2	Expression management<br>Patient's hygiene	Need to learn expression management.

‘We try not to show disgust we only guide them for hygiene. So, they can protect themselves from disease.’<br>‘It is not the matter of poverty that the patients present themselves in poor hygiene. It is about their preference to not to spent their money on a soap and buying talk time for their mobile phone of more worth...’				
